# Increased Risk of Thyroid Cancer in Patients with Graves’ Disease and Thyroid Nodules: A Retrospective Analysis

**DOI:** 10.3390/diagnostics16142260

**Published:** 2026-07-20

**Authors:** Mihaela Andreea Precup, Flaviu Ionut Faur, Marco Marian, Paul Pasca, Draga-Maria Mandi, Dan Brebu, Andrei Korodi, Cosmin Mihai Burta, Amadeus Dobrescu, Ciprian Duta

**Affiliations:** 1 Doctoral School, Victor Babes University of Medicine and Pharmacy Timisoara, E. Murgu Square, No. 2, 300041 Timisoara, Romania; mihaela.precup@umft.ro (M.A.P.); paul.pasca@umft.ro (P.P.); mihai.burta@umft.ro (C.M.B.); 2 Researching Future Surgery II Research Center, Department X, Discipline of General Surgery II, Faculty of Medicine, Victor Babes University of Medicine and Pharmacy Timisoara, E. Murgu Square, No. 2, 300041 Timisoara, Romania; flaviu.faur@umft.ro (F.I.F.); marian.marco@umft.ro (M.M.); dobrescu.amadeus@umft.ro (A.D.); duta.ciprian@umft.ro (C.D.); 3 General Surgery Department, Coltea Clinical Hospital, 030171 Bucharest, Romania; 4 2nd Surgery Clinic, Emergency Clinical County Hospital of Arad, 2-4 Andreny Karoly Str., 310037 Arad, Romania; 5 Department of Medicine, Faculty of Medicine, “Vasile Goldis” Western University of Arad, L. Rebreanu St. 86, 310048 Arad, Romania

**Keywords:** Graves’ disease, thyroid nodules, thyroid cancer, papillary thyroid carcinoma, malignancy risk, FNA, risk stratification

## Abstract

**Background:** Graves’ disease (GD) is an autoimmune disorder frequently associated with thyroid nodules, raising concerns regarding malignancy risk. However, reported cancer prevalence varies widely, and data from Eastern European populations remain limited. This study aimed to evaluate the risk of thyroid cancer in GD patients and to identify clinical predictors of malignancy. **Methods:** We conducted a retrospective cohort study including 168 patients diagnosed with GD between 2018 and 2022 at a tertiary referral center. Clinical, ultrasonographic, and histopathological data were analyzed. Associations between thyroid nodules and malignancy were assessed using chi-square tests and logistic regression analysis. **Results:** Thyroid nodules were identified in a substantial proportion of GD patients, and among patients with thyroid nodules who underwent thyroidectomy, thyroid carcinoma was confirmed by histopathological examination in 11 of 42 cases (26.2%). The presence of nodules was significantly associated with malignancy (OR = 4.337, 95% CI: 1.528–7.146, *p* < 0.001). Increasing age was independently associated with higher cancer risk (r = 0.221, *p* = 0.004). Papillary thyroid carcinoma (PTC) was the predominant histological subtype, most commonly presenting as early-stage disease (pT1aNxR0). **Conclusions:** Thyroid nodules were significantly associated with thyroid malignancy in patients with Graves’ disease. In this tertiary referral cohort, papillary thyroid carcinoma was the predominant histological subtype, supporting careful ultrasound evaluation and appropriate cytological assessment of thyroid nodules.

## 1. Introduction

Graves’ disease (GD) is the most common cause of autoimmune hyperthyroidism and is characterized by the presence of thyroid-stimulating immunoglobulins that activate the thyrotropin receptor, leading to excessive thyroid hormone production and diffuse thyroid hyperplasia [1]. In addition to hyperthyroidism, GD is associated with a broad spectrum of thyroid morphological abnormalities, including diffuse goiter and focal nodular disease. Although thyroid nodules are frequently encountered in the general population, their presence in patients with GD has attracted particular clinical interest because of the potentially increased risk of thyroid malignancy [2,3,4]. The coexistence of thyroid nodules and Graves’ disease represents a major diagnostic and therapeutic challenge in endocrine practice [5]. Previous studies have reported that approximately 20–25% of patients with GD develop thyroid nodules, with prevalence increasing with age, disease duration, iodine intake, and improved imaging accessibility. Importantly, unlike nodules detected in euthyroid individuals, nodules arising in the context of GD appear to carry a higher probability of malignancy. Several observational studies and recent meta-analyses have demonstrated an increased incidence of thyroid carcinoma in patients with GD, particularly papillary thyroid carcinoma (PTC), the most common histological subtype of differentiated thyroid cancer [6,7,8].

The pathophysiological mechanisms underlying the association between GD and thyroid carcinogenesis remain incompletely understood. Chronic autoimmune stimulation, sustained thyrotropin receptor activation, oxidative stress, and increased thyroid cell proliferation have all been proposed as potential contributors to malignant transformation [9]. Furthermore, thyroid-stimulating antibodies may exert trophic effects on follicular epithelial cells, promoting tumor growth and angiogenesis. In addition, the inflammatory microenvironment associated with autoimmune thyroid disease may contribute to genomic instability and carcinogenesis through cytokine-mediated signaling pathways [10]. Advances in thyroid imaging have substantially improved the detection of nodular disease in patients with GD. High-resolution ultrasonography remains the cornerstone of thyroid nodule evaluation and provides essential information regarding nodule size, echogenicity, margins, calcifications, shape, and vascularization patterns. Contemporary risk stratification systems, including the Thyroid Imaging Reporting and Data System (TI-RADS), have enhanced the ability to identify nodules with suspicious ultrasonographic features. Additional imaging modalities such as Doppler ultrasonography and elastography may further improve diagnostic performance by evaluating vascular architecture and tissue stiffness [11,12,13,14]. Nevertheless, fine-needle aspiration (FNA) biopsy continues to represent the gold standard for differentiating benign from malignant thyroid lesions. Despite increasing awareness of the association between GD and thyroid cancer, considerable heterogeneity persists across published studies regarding the true magnitude of malignancy risk. Reported prevalence rates vary widely depending on study design, geographic region, patient selection, and surgical indications. Moreover, most available evidence derives from surgically treated populations, potentially introducing selection bias and overestimating cancer prevalence [15,16,17,18,19,20,21,22]. Data from Eastern European populations remain relatively limited, further emphasizing the need for additional regional studies evaluating the clinical behavior of thyroid nodules in GD patients [23,24,25,26]. Understanding the relationship between GD, thyroid nodules, and thyroid cancer is of considerable clinical importance because early identification of malignant lesions may significantly influence therapeutic strategies, surgical decision-making, and long-term prognosis. Accurate risk stratification is essential to avoid unnecessary invasive procedures while ensuring timely diagnosis of clinically significant malignancies [27,28].

Therefore, the aim of the present study was to evaluate the risk of thyroid cancer in patients with Graves’ disease and thyroid nodules and to identify clinical factors associated with malignancy in a retrospective cohort from a tertiary referral center.

## 2. Materials and Methods

### 2.1. Study Design and Population

This retrospective observational cohort study was conducted at the “Pius Brînzeu” County Emergency Hospital, Timișoara, Romania, between January 2018 and December 2022. The study included patients diagnosed with Graves’ disease (GD) who underwent clinical, biochemical, and ultrasonographic evaluation during the study period.

The diagnosis of Graves’ disease was established based on clinical findings consistent with hyperthyroidism, suppressed thyroid-stimulating hormone (TSH) levels, elevated thyroid hormone concentrations, and thyroid-stimulating immunoglobulin positivity when available in the medical records.

Patients were stratified according to the presence or absence of thyroid nodules detected by cervical ultrasonography. The present investigation was designed as a retrospective cohort analysis focusing on the association between thyroid nodules and histopathologically confirmed thyroid malignancy in patients with Graves’ disease.

Because the study was conducted in a tertiary referral center, a proportion of patients were referred for specialized endocrine or surgical evaluation. Consequently, the study population should be interpreted as a selected tertiary-care cohort rather than a population-based sample.

### 2.2. Inclusion Criteria

- Diagnosis of Graves’ disease established according to clinical, biochemical, and imaging criteria;
- Available thyroid ultrasound examination;
- Complete clinical records;
- Available histopathological reports for patients who underwent thyroid surgery.

Both adolescent and adult patients were eligible for inclusion if a confirmed diagnosis of Graves’ disease was available.

### 2.3. Exclusion Criteria

- Previous thyroid malignancy diagnosed before Graves’ disease;
- Previous thyroid surgery;
- Incomplete clinical records;
- Insufficient diagnostic information to confirm Graves’ disease.

### 2.4. Data Collection

Data were extracted from electronic medical records and included:

- Demographics (age, sex);
- Ultrasound findings (presence/absence of nodules);
- Histopathological diagnosis (when available).

Thyroid nodules were identified using cervical ultrasonography performed as part of routine endocrine evaluation. Due to the retrospective nature of the study, detailed ultrasonographic descriptors, including TI-RADS classification, echogenicity, microcalcifications, margin characteristics, vascularity patterns, and Bethesda cytology categories, were not consistently available for all patients and therefore could not be included in the final statistical analysis. Histopathological diagnosis was considered the reference standard whenever surgical specimens were available. Patients with thyroid nodules who did not undergo surgery had no histopathological confirmation available and were therefore not included in the calculation of histopathologically confirmed thyroid cancer prevalence.

### 2.5. Statistical Analysis

Statistical analysis was performed using SPSS (version 20).

- Continuous variables were expressed as mean ± standard deviation;
- Categorical variables were expressed as percentages;
- Associations were assessed using chi-square tests;
- Logistic regression was used to estimate odds ratios (ORs);
- Correlations were evaluated using Pearson correlation coefficients.

A *p*-value < 0.05 was considered statistically significant.

### 2.6. Ethics Approval

This study was conducted in accordance with the ethical principles outlined in the Declaration of Helsinki and approved by the Ethics Committee of the “Pius Brînzeu” County Emergency Hospital, Timișoara (No. 176/11.04.2024). Due to the retrospective nature of the study and the use of anonymized clinical data, the requirement for informed patient consent was waived by the institutional review board. All patient data were handled confidentially and processed in compliance with applicable data protection regulations.

## 3. Results

### 3.1. Demographic and Clinical Characteristics

A total of 168 patients with Graves’ disease were included in the study. Thyroid nodules were identified in 55 patients (32.7%), of whom 42 subsequently underwent thyroidectomy according to clinical indications. The mean age of the cohort was 49.32 years (SD ± 14.31), with ages ranging from 15 to 81 years. Table 1 summarizes the descriptive statistics for patient age across the study period (2018–2022). The overall mean age of patients diagnosed with Graves’ disease was relatively stable throughout the analyzed years, ranging from 48.87 years in 2019 to 55.00 years in 2018. Standard deviation values between 13.25 and 16.22 years indicate moderate variability in age distribution within the cohort.

The study population included both younger and older individuals, with ages ranging from 15 to 81 years. Median age values remained close to 50 years during most years of the study, suggesting that middle-aged adults represented the predominant affected population. Interquartile ranges demonstrated a relatively broad age distribution, with the 25th percentile generally situated between 34 and 46 years and the 75th percentile between 55 and 65 years. These findings indicate that Graves’ disease associated with thyroid nodules and malignancy occurred predominantly in middle-aged and older adults. Older patients represented a substantial proportion of the study population (Table 2).

**Figure 1:**
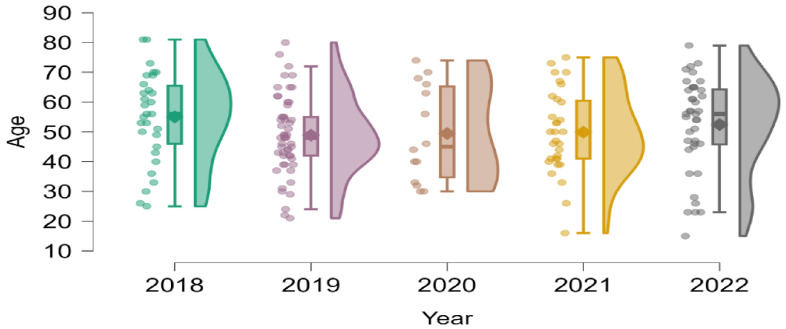
Raincloud plot for age distribution.

Figure 1 presents a raincloud plot illustrating the age distribution of patients with Graves’ disease across the study period (2018–2022). This graphical representation combines density distribution, boxplot visualization, and individual data points, providing a comprehensive overview of age variability within the cohort.

The plot demonstrates a relatively similar age distribution across all years, with most patients clustered between 40 and 65 years of age. The median age remained close to 50 years throughout the study period, consistent with the descriptive statistical analysis. The density curves indicate a predominance of middle-aged and older adult patients, while the spread of individual observations highlights moderate interindividual variability. Additionally, the plot reveals the presence of several younger and older outliers, reflecting the broad age range of the study population. Slight shifts in distribution patterns between years may suggest temporal differences in patient presentation; however, no major deviations in age structure were observed during the analyzed period. Overall, the raincloud plot visually supports the observation that Graves’ disease associated with thyroid nodules and malignancy predominantly affects middle-aged and older individuals, reinforcing the importance of age as a potential factor associated with increased thyroid cancer risk.

### 3.2. Prevalence of Thyroid Nodules and Cancer

**Figure 2:**
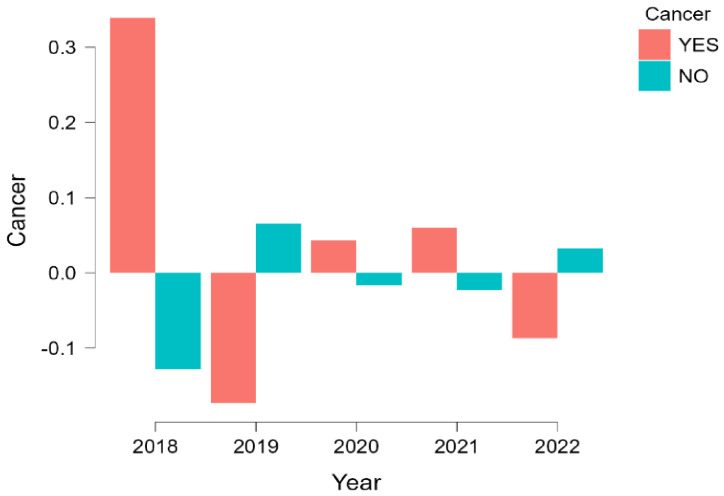
Thyroid nodule and cancer prevalence by year.

Table 3 and Figure 2 summarize the frequency of histopathologically confirmed thyroid cancer among surgically treated patients with Graves’ disease between 2018 and 2022. Of the 42 patients with thyroid nodules who underwent thyroidectomy, thyroid carcinoma was confirmed in 11 cases (26.2%). The reported prevalence therefore reflects the surgically treated subgroup and should not be interpreted as the prevalence among all patients with Graves’ disease and thyroid nodules. These findings indicate that thyroid nodules were frequently identified in patients with Graves’ disease throughout the study period.

Table 3 and Figure 2 summarize the yearly frequency and prevalence of thyroid cancer among patients diagnosed with Graves’ disease during the 2018–2022 study period. The results demonstrate that thyroid malignancy was identified in a substantial proportion of patients throughout all analyzed years. The highest prevalence of thyroid cancer was recorded in 2018, when 36.7% of patients were diagnosed with malignancy, whereas the lowest prevalence was observed in 2019 (22.6%). In the subsequent years, cancer prevalence remained relatively stable, ranging between 25.0% and 29.0%. Overall, although most patients did not develop thyroid cancer, the proportion of malignant cases remained clinically significant across the entire cohort.

The relationship between thyroid nodules and cancer was analyzed using chi-square tests (Table 4).

Table 4 presents the results of the chi-square analysis performed to evaluate the association between thyroid nodules and thyroid cancer in patients with Graves’ disease across the study period. The analysis demonstrated a statistically significant association between the presence of thyroid nodules and malignancy in most analyzed years, as well as in the overall cohort. Significant associations were identified in 2018 (Χ^2^ = 8.684, *p* = 0.003), 2019 (Χ^2^ = 11.009, *p* < 0.001), and 2022 (Χ^2^ = 6.420, *p* = 0.011), indicating that patients with thyroid nodules had a significantly higher likelihood of developing thyroid cancer during these years. In 2021, the association approached statistical significance (Χ^2^ = 3.699, *p* = 0.054), while no significant association was observed in 2020 (Χ^2^ = 1.527, *p* = 0.217), most likely due to the smaller sample size included during that year.

Importantly, the pooled analysis of all patients demonstrated a highly significant overall association between thyroid nodules and thyroid malignancy (Χ^2^ = 30.831, *p* < 0.001). The linear regression analysis (Table 5) demonstrated that patients with thyroid nodules and those diagnosed with thyroid cancer were significantly older than patients without these conditions. The R^2^ value of 0.128 suggests that the model explains 12.8% of the variation in age, indicating that other factors may contribute to the observed differences.

The linear regression model was constructed with age as the dependent variable and thyroid nodules and cancer status as explanatory variables. The adjusted model explained 12.8% of the observed variability in age (R^2^ = 0.128), indicating a statistically significant association between age and the presence of nodules and malignancy.

Furthermore, the reduction in the root mean square error (RMSE) from 14.647 in the null model to 13.757 in the adjusted model indicates an improved model fit after inclusion of the predict. These findings indicate that patients presenting thyroid nodules and malignancy tended to be older than patients without these conditions.

Overall, these findings indicate that increasing age was associated with both thyroid nodule presence and thyroid cancer diagnosis in this cohort.

**Figure 3:**
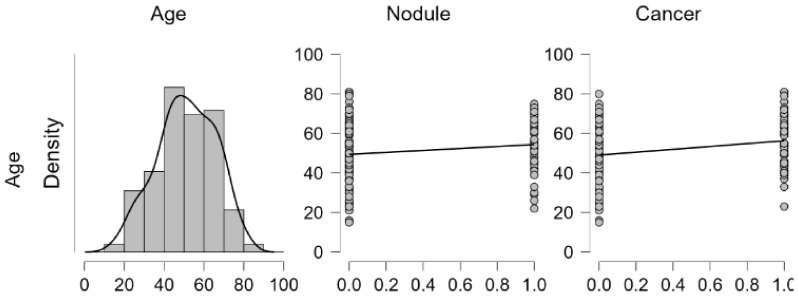
ANOVA for age, nodules, and cancer.

- The ANOVA test (Table 6, Figure 3) was conducted to examine the impact of age, nodule presence, and cancer diagnosis on variance across groups. The results showed a significant difference (F = 12.153, *p* < 0.001), suggesting that age influences the likelihood of thyroid nodules and cancer.

**Table 6:** ANOVA evaluating the effect of thyroid nodules and thyroid cancer on age.

ANOVA						
Model		Sum of Squares	df	Mean Square	F	*p*
M_1_	Regression	4600.262	2	2300.131	12.153	<0.001
	Residual	31,227.357	165	189.257		
	Total	35,827.619	167			

Note. M_1_ includes Nodule and Cancer. Note. The intercept model is omitted, as no meaningful information can be shown.

The regression coefficients demonstrated that patients with thyroid nodules were significantly older than patients without nodules (β = 9.713, *p* < 0.001). Similarly, patients diagnosed with thyroid cancer were significantly older than patients without a malignancy (β = 11.618, *p* < 0.001) (Table 7).

### 3.3. Odds Ratio and Logistic Regression Analysis

The odds ratio (OR) for thyroid cancer in patients with nodules was 4.337 (95% CI: 1.528–7.146, *p* < 0.001), indicating a strong association (Table 8).

Linear regression analysis identified significant associations between age and both thyroid nodule presence and thyroid cancer diagnosis. Patients with nodules and malignancy were significantly older than those without these conditions.

### 3.4. Correlation Analysis

Pearson correlation analysis (Table 9) revealed a weak but significant positive correlation between age and thyroid cancer (r = 0.221, *p* = 0.004), suggesting an increased malignancy risk in older patients. Additionally, a moderate negative correlation between nodules and cancer (r = −0.428, *p* < 0.001), reflecting the binary coding of the analyzed variables rather than an inverse biological association between thyroid nodules and thyroid cancer.

### 3.5. Histopathological Findings

The distribution of tumor staging is summarized in Table 10 and predominantly identified papillary thyroid carcinoma (PTC), with the pT1aNxR0 subtype being the most common.

Papillary thyroid carcinoma represented the predominant histopathological subtype identified throughout the study period. Most malignant lesions corresponded to low-stage tumors, with pT1aNxR0 representing the most frequently encountered pathological category. 

## 4. Discussion

The present study demonstrated a significant association between thyroid nodules and thyroid malignancy in patients with Graves’ disease (GD), reinforcing the growing body of evidence suggesting that nodular thyroid disease in the setting of autoimmune hyperthyroidism should be approached with increased oncologic vigilance. Our findings showed that thyroid cancer was diagnosed in 26.2% of patients with thyroid nodules who underwent surgical treatment and histopathological evaluation within this tertiary-care cohort, while the presence of nodules was associated with a more than fourfold increased risk of malignancy (OR = 4.337, *p* < 0.001). These results are consistent with previously published meta-analyses and observational studies reporting substantially higher malignancy rates in GD patients compared with euthyroid populations [2,3,8,10].

Importantly, the malignancy prevalence observed in the present study should be interpreted in the context of a tertiary referral cohort. Because histopathological confirmation was available primarily in surgically treated patients, the reported prevalence reflects the malignancy frequency among selected patients undergoing surgical evaluation and should not be interpreted as the prevalence of thyroid cancer among all patients with Graves’ disease. Accordingly, the reported 26.2% prevalence should be interpreted as the prevalence observed among surgically treated patients within a tertiary referral center rather than the intrinsic malignancy risk of all patients with Graves’ disease and thyroid nodules.

The relationship between GD and thyroid carcinogenesis remains incompletely elucidated; however, several pathophysiological mechanisms have been proposed. Chronic autoimmune stimulation of the thyroid gland may contribute to increased cellular proliferation, oxidative stress, and genomic instability, thereby creating a microenvironment favorable for malignant transformation. Thyroid-stimulating immunoglobulins (TSIs), which play a central role in GD pathogenesis, may exert trophic effects on thyroid follicular cells through persistent activation of the TSH receptor. This continuous stimulation has been hypothesized to promote both thyroid hyperplasia and neoplastic progression. Additionally, inflammatory cytokines and chemokines, including IL-6, TNF-α, and CXCL10, have been implicated in both autoimmune thyroid disease and thyroid carcinogenesis, potentially promoting tumor initiation, angiogenesis, and tumor progression through chronic immune activation [1,3,9].

Our observed malignancy prevalence falls within the upper range reported in previous studies. Staniforth et al. demonstrated in a meta-analysis that thyroid carcinoma occurs more frequently in patients with Graves’ disease than previously assumed, particularly when thyroid nodules are present [10]. Similarly, Papanastasiou et al. reported a significantly increased risk of malignancy among surgically treated Graves’ disease patients with coexisting nodular disease [2]. The relatively elevated prevalence observed in our cohort may partly reflect the tertiary referral nature of our institution, where patients with more complex thyroid pathology are more likely to be evaluated and surgically managed. Papillary thyroid carcinoma (PTC) represented the predominant histopathological subtype in our cohort, with most tumors corresponding to early-stage lesions, particularly pT1aNxR0. These findings are in agreement with current literature demonstrating that PTC is the most frequent thyroid malignancy associated with GD. The predominance of microcarcinomas and low-stage tumors may reflect the increasing use of high-resolution ultrasonography and the implementation of more rigorous thyroid surveillance strategies in patients with autoimmune thyroid disorders. Earlier detection may contribute to improved prognosis and reduced disease-specific mortality [4,11,27].

Age emerged as a significant factor associated with malignancy risk in our analysis. Pearson correlation analysis demonstrated a weak but statistically significant positive association between increasing age and thyroid cancer occurrence (r = 0.221, *p* = 0.004). Furthermore, regression analyses indicated that patients diagnosed with thyroid nodules and malignancy were significantly older than those without these conditions. These findings support previous evidence suggesting that advanced age may contribute to a more aggressive thyroid disease phenotype and increased cancer susceptibility. From a clinical perspective, this observation highlights the importance of individualized surveillance protocols, particularly in older GD patients presenting suspicious thyroid nodules [2,4,25].

The clinical implications of our findings are considerable. Although diffuse hypervascular thyroid enlargement is a characteristic feature of GD, the coexistence of focal nodular disease should not be underestimated. Ultrasound evaluation represents an important component of the diagnostic assessment of patients with Graves’ disease, particularly in those presenting thyroid nodules, palpable abnormalities, or other clinical features raising suspicion for malignancy. Particular attention should be directed toward nodules exhibiting suspicious ultrasonographic characteristics, including hypoechogenicity, irregular margins, microcalcifications, taller-than-wide morphology, and increased intranodular vascularization [18,19,20,21]. In such cases, timely fine-needle aspiration (FNA) biopsy is crucial for appropriate cytological assessment and therapeutic planning. In addition, our results support the growing role of standardized ultrasound risk stratification systems, such as ACR TI-RADS and EU-TIRADS, in improving malignancy prediction in thyroid nodules associated with GD. Incorporating these systems into routine endocrine practice may facilitate earlier identification of clinically significant lesions while reducing unnecessary invasive procedures for low-risk nodules [20,21,23].

The present study possesses several important strengths. First, the study included a relatively well-defined cohort of patients diagnosed with GD over a five-year period. Second, histopathological confirmation was available in malignant cases, increasing diagnostic accuracy and reliability of the reported outcomes. Third, multiple statistical approaches, including chi-square analysis, regression models, and correlation analyses, were used to comprehensively evaluate the relationship between nodules, age, and malignancy risk. Furthermore, the study contributes valuable regional data from an Eastern European tertiary center, addressing an area currently underrepresented in the literature. Nevertheless, several limitations should be acknowledged. The retrospective design inherently introduces the possibility of selection bias and limits causal inference. Additionally, the study was conducted in a single tertiary referral center, which may reduce generalizability to broader populations and potentially overestimate malignancy prevalence due to referral patterns. The absence of long-term follow-up data prevented evaluation of recurrence rates, disease progression, and long-term oncologic outcomes. Another important limitation is the lack of molecular characterization of thyroid tumors, including BRAF, RAS, RET/PTC, or TERT promoter mutations, which are increasingly recognized as important prognostic and diagnostic biomarkers in differentiated thyroid carcinoma.

Future studies should therefore focus on prospective multicenter designs integrating ultrasonographic, cytological, histopathological, and molecular parameters in order to develop more accurate risk stratification models for GD-associated thyroid nodules. Additionally, investigating the molecular interplay between thyroid autoimmunity and carcinogenesis may provide further insights into the mechanisms linking GD and thyroid cancer development.

Additional limitations include the absence of standardized TI-RADS classifications, Bethesda cytology categories, TRAb titers, and detailed ultrasonographic characteristics for all patients. Furthermore, patients with thyroid nodules managed conservatively without surgery could not be included in the histopathological analysis, which may have contributed to selection bias.

Furthermore, standardized TI-RADS classification, Bethesda cytology categories, and detailed ultrasonographic descriptors were not consistently available because of the retrospective study design. Consequently, the influence of ultrasound-based risk stratification on surgical decision-making could not be evaluated and may have contributed to selection bias.

## 5. Conclusions

In conclusion, thyroid nodules were significantly associated with thyroid malignancy in this tertiary-care cohort of patients with Graves’ disease. However, the reported malignancy prevalence reflects surgically treated patients with histopathological confirmation and should not be generalized to the entire Graves’ disease population. Careful ultrasound evaluation and appropriate cytological assessment remain essential for identifying patients at increased risk of malignancy. Prospective multicenter studies incorporating standardized imaging, cytological, and molecular data are warranted to further refine risk stratification and optimize clinical decision-making.

## Figures and Tables

**Table 1:** Descriptive statistics for age.

Descriptive Statistics					
	Age				
	2018	2019	2020	2021	2022
Mean	55.000	48.868	49.429	49.871	52.450
Std. Deviation	15.191	13.252	16.223	13.983	15.883
Range	56.000	59.000	44.000	59.000	64.000
Minimum	25.000	21.000	30.000	16.000	15.000
Maximum	81.000	80.000	74.000	75.000	79.000
25th percentile	46.000	42.000	34.750	41.000	45.750
50th percentile	56.000	48.000	45.000	50.000	56.000
75th percentile	65.500	55.000	65.250	60.500	64.250

**Table 2:** Nodule prevalence.

Year	Nodule	Frequency	Percent	Valid Percent	Cumulative Percent
2018	NO	20	66.667	66.667	66.667
	YES	10	33.333	33.333	100.000
	Missing	0	0.000		
	Total	30	100.000		
2019	NO	31	58.491	58.491	58.491
	YES	22	41.509	41.509	100.000
	Missing	0	0.000		
	Total	53	100.000		
2020	NO	11	78.571	78.571	78.571
	YES	3	21.429	21.429	100.000
	Missing	0	0.000		
	Total	14	100.000		
2021	NO	24	77.419	77.419	77.419
	YES	7	22.581	22.581	100.000
	Missing	0	0.000		
	Total	31	100.000		
2022	NO	27	67.500	67.500	67.500
	YES	13	32.500	32.500	100.000
	Missing	0	0.000		
	Total	40	100.000		

**Table 3:** Cancer prevalence.

Year	Cancer	Frequency	Percent	Valid Percent	Cumulative Percent
2018	YES	11	36.667	36.667	36.667
	NO	19	63.333	63.333	100.000
	Missing	0	0.000		
	Total	30	100.000		
2019	YES	12	22.642	22.642	22.642
	NO	41	77.358	77.358	100.000
	Missing	0	0.000		
	Total	53	100.000		
2020	YES	4	28.571	28.571	28.571
	NO	10	71.429	71.429	100.000
	Missing	0	0.000		
	Total	14	100.000		
2021	YES	9	29.032	29.032	29.032
	NO	22	70.968	70.968	100.000
	Missing	0	0.000		
	Total	31	100.000		
2022	YES	10	25.000	25.000	25.000
	NO	30	75.000	75.000	100.000
	Missing	0	0.000		
	Total	40	100.000		

**Table 4:** Chi-Squared Tests.

Year		Value	df	*p*
2018	Χ^2^	8.684	1	0.003
N	30			
2019	Χ^2^	11.009	1	<0.001
N	53			
2020	Χ^2^	1.527	1	0.217
N	14			
2021	Χ^2^	3.699	1	0.054
N	31			
2022	Χ^2^	6.420	1	0.011
N	40			
Total	Χ^2^	30.831	1	<0.001
N	168			

**Table 5:** Linear Regression analysis between age, thyroid nodules and thyroid cancer.

Model Summary—Age				
Model	R	R^2^	Adjusted R^2^	RMSE
M_0_	0.000	0.000	0.000	14.647
M_1_	0.358	0.128	0.118	13.757

Note. M_1_ includes Nodule and Cancer.

**Table 7:** Linear Regression Coefficients Assessing the Association Between Age, Thyroid Nodules, and Thyroid Cancer in Patients with Graves’ Disease.

Model		Unstandardized	Standard Error	Standardized	t	*p*
M_0_	(Intercept)	51.048	1.130		45.173	<0.001
M_1_	(Intercept)	44.687	1.681		26.588	<0.001
	Nodule	9.713	2.503	0.312	3.880	<0.001
	Cancer	11.618	2.634	0.355	4.410	<0.001

**Table 8:** Odds Ratio Analysis of Thyroid Cancer Risk Associated with Thyroid Nodules in Patients with Graves’ Disease.

			95% Confidence Intervals		
Year		Odds Ratio (OR)	Lower	Upper	*p*
2018	Odds ratio	3.236	0.271	6.200	
	Fisher’s exact test	∞	0.666	∞	0.004
2019	Odds ratio	3.362	0.471	6.253	
	Fisher’s exact test	∞	0.988	∞	<0.001
2020	Odds ratio	1.435	1.750	4.620	
	Fisher’s exact test	∞	1.859	∞	0.505
2021	Odds ratio	2.219	0.756	5.193	
	Fisher’s exact test	∞	0.418	∞	0.077
2022	Odds ratio	2.785	0.140	5.710	
	Fisher’s exact test	∞	0.307	∞	0.016
Total	Odds ratio	4.337	1.528	7.146	
	Fisher’s exact test	∞	2.216	∞	<0.001

**Table 9:** Pearson Correlation Analysis Between Age, Thyroid Nodules, and Thyroid Cancer in Patients with Graves’ Disease.

Variable		Age	Nodule	Cancer
1. Age	Pearson’s r	—		
	*p*-value	—		
2. Nodule	Pearson’s r	0.160	—	
	*p*-value	0.038	—	
3. Cancer	Pearson’s r	0.221	−0.428	—
	*p*-value	0.004	<0.001	—

**Table 10:** Frequencies of histological diagnoses.

Year	Diagnosis	Frequency	Percent	Valid Percent	Cumulative Percent
2018	pT1bN0R0	1	3.333	9.091	9.091
	pT1NxMxR0	1	3.333	9.091	18.182
	pT2 NxR0	1	3.333	9.091	27.273
	pT1aNxR0	4	13.333	36.364	63.636
	pT2NxR0	1	3.333	9.091	72.727
	pT1aN0R0	1	3.333	9.091	81.818
	pT1bNxMxL	1	3.333	9.091	90.909
	pT3aL	1	3.333	9.091	100.000
	Total	11	100		
2019	pT1bN0R0	1	1.887	11.111	11.111
	pT1aNx	2	3.774	22.222	33.333
	Nx	1	1.887	11.111	44.444
	pT1Nx	1	1.887	11.111	55.556
	pT1bN0	1	1.887	11.111	66.667
	pT1a NxR0	1	1.887	11.111	77.778
	pT1b N0	1	1.887	11.111	88.889
	pT3aN0R0	1	1.887	11.111	100.000
	Total	9	100		
2020	pT1aNxR0	1	7.143	25.000	25.000
	pT1aNx	1	7.143	25.000	50.000
	pT1a NxR0	1	7.143	25.000	75.000
	pT1a N0	1	7.143	25.000	100.000
	Total	4	100		
2021	pT1aNxR0	3	9.677	37.500	37.500
	pT1bNxL1	1	3.226	12.500	50.000
	pT1NxR0	2	6.452	25.000	75.000
	pT1bNxL	1	3.226	12.500	87.500
	pT1aNxV1R0	1	3.226	12.500	100.000
	Total	8	100.000		
2022	pT1aNxR0	4	10.000	40.000	40.000
	pT1bNxL	2	5.000	20.000	60.000
	pT2N0	1	2.500	10.000	70.000
	pT1aN1L1	1	2.500	10.000	80.000
	pT2NxMxL	1	2.500	10.000	90.000
	pT1aNxL	1	2.500	10.000	100.000
	Total	10	100.000		

TNM = Tumor–Node–Metastasis classification system; Nx indicates that regional lymph nodes were not assessed; Rx denotes that resection margin status could not be evaluated.

## Data Availability

The data presented in this study are available from the corresponding author upon reasonable request. The data are not publicly available due to privacy and institutional restrictions.
